# Factors Associated with Intention of Serbian Public Health Workers to Leave the Job: A Cross-Sectional, Population-Based Study

**DOI:** 10.3390/ijerph182010652

**Published:** 2021-10-11

**Authors:** Katica Tripković, Milena Šantrić-Milićević, Milena Vasić, Mirjana Živković-Šulović, Marina Odalović, Vesna Mijatović-Jovanović, Zoran Bukumirić

**Affiliations:** 1 Department for Analysis, Planning and Organization of Health Care, City Institute of Public Health Belgrade, 11000 Belgrade, Serbia; 2 Centre–School of Public Health and Health Management, Faculty of Medicine, University of Belgrade, 11000 Belgrade, Serbia; milena.santric-milicevic@med.bg.ac.rs; 3 Faculty of Medicine, Institute of Social Medicine, University of Belgrade, 11000 Belgrade, Serbia; 4 Faculty of Dentistry Pancevo, University Business Academy in Novi Sad, 26000 Pancevo, Serbia; milena_vasic@batut.org.rs; 5 Institute of Public Health of Serbia “Dr Milan Jovanović Batut”, 11000 Belgrade, Serbia; mirjana_sulovic@batut.org.rs; 6 Department of Social Pharmacy and Pharmaceutical Legislation, Faculty of Pharmacy, University of Belgrade, 11000 Belgrade, Serbia; marina.odalovic@pharmacy.bg.ac.rs; 7 Faculty of Medicine, University of Novi Sad, 21000 Novi Sad, Serbia; vesna.mijatovic-jovanovic@mf.uns.ac.rs; 8 Institute of Public Health of Vojvodina, 21000 Novi Sad, Serbia; 9 Faculty of Medicine, Institute of Medical Statistics and Informatics, University of Belgrade, 11000 Belgrade, Serbia; zoran.bukumiric@med.bg.ac.rs

**Keywords:** public health workforce, intention to leave job, retention, public health institutes

## Abstract

Recruitment and retention of public health workers (PHWs) is crucial for the optimal functioning of the public health system at a time of budget cuts and the threat of a pandemic. Individual and job-related variables were examined by univariate and multivariate logistic regression to identify predictors of the intention to leave a job during the COVID-19 outbreak among Serbian PHWs in 25 institutes of public health (*n* = 1663 respondents, of which 73.1% were female). A total of 20.3% of PHWs intended to leave their current job within the next five years. Males and persons aged younger than 55 years who had additional practice were more likely to report an intention to leave their job than females, those older than 54 years and those without additional work. While uncertainty and fear of infection during the COVID-19 pandemic were almost perceived as job attractiveness, other job-related characteristics were identified as significant barriers to maintaining the sufficient capacity of qualified PHWs in the future. Authorities need to address these factors, including the following: the feeling of tension, stress or pressure, and unavailability of information during the COVID-19 pandemic, as well as dissatisfaction with respect, valuation, and the job in general.

## 1. Introduction

Effective delivery of essential public health services [1] requires the availability and adequate distribution of a highly skilled and capable public health workforce [2,3]. However, current environmental, demographic, economic, and political developments suggest an increase in the future severity of public health threats, such as a growing burden of chronic diseases and social and health inequalities, natural and artificial disasters and emergencies, antimicrobial resistance, etc. [3,4,5,6,7]. Additionally, a threat posed by infective disease outbreaks such as the COVID-19 pandemic illustrates their severity and devastating impact [4,5,8,9,10]. Therefore, the need for effective response to both identified and emerging public health threats by strengthening the capacity of the public health workforce, and thus public health as a whole [8], is obvious. Public health systems have been chronically underfunded and understaffed, even in wealthy countries such as the USA, Canada, and Australia, and even more in much of the developing world [3,11,12,13]. These problems exacerbate numerous additional public health workforce challenges, including the variety of definitions of public health workers, aging of the workforce, inconsistencies in educational approaches and opportunities, and skill shortages. The increasing international recruitment of health workers poses difficulties for retaining skilled professionals within the public health workforce and the deployment of adequate substitutes [14,15,16].

Intention to leave is defined as “an individual’s own estimated probability of leaving the organization or profession within a specific period” [17,18]. In line with Ajzen’s Theory of Planned Behavior [19] and substantiated by Mobley’s Model of the Turnover Process [20], intention to leave is considered one of the most important predictors of actual turnover. The Turnover Process starts with an individual’s intention to leave a job after assessing the current job, accompanied by a feeling of dissatisfaction. An evaluation of the pros and cons of leaving a job and consideration of alternatives can finally result in actual turnover. At the same time, numerous other factors, most often grouped as individual, organizational, and external (contextual), influence the intention to leave a job [21,22]. During the employees’ decision process to leave [20], the organization can proactively react and manage the turnover process effectively [23,24] by mainly addressing the individual and organizational factors. Intention to leave a job may be a critical and increasing problem in healthcare settings since it could contribute to the already existing and forecasted health workforce shortage and jeopardize the overall health systems’ performance [25,26,27]. Several factors influence the intention to leave the unit, organization, and profession, including dissatisfaction with the work environment, pay, interpersonal relationships, professional improvement opportunities, and individual (e.g., age, gender, occupation) and organizational factors (e.g., management, leadership, work stress) [25,26,27,28,29,30]. These factors were the focus of this study.

In Serbia, institutes of public health (IPHs) are understaffed and endure inadequate workforce distribution across its districts [31,32], perhaps due to aging and low recruitment of new professionals, insufficient attractiveness of the profession, and budget cuts [31,32,33,34,35]. Previous studies in Serbia have mainly studied the intention to work abroad among physicians, nurses, and medical students [36,37,38]. In this paper, we considered of immense importance examination of the intention to leave among workers in public health (PHWs), as such studies are rare in countries other than the USA [39,40,41,42,43,44,45]. Assuming that hard work during the COVID-19 pandemic can affect health workers’ commitment to work [46], this study aimed to examine individual and job-related predictors of the intention to leave a current job among Serbian PHWs during the COVID-19 outbreak.

## 2. Materials and Methods

### 2.1. The Setting, Study Design, and Participants

This study applied a cross-sectional design to explore the intention to leave a current job within the next five years in a population of PHWs of Serbia in 2020 during the COVID-19 pandemic. According to a recent systematic review [47], most concepts of PHWs define workers based on their occupation or their place of work. With this in mind, the examined population of PHWs in this study consisted of employees in institutes of public health in the Republic of Serbia.

The study design is a secondary analysis of the selected variables from the 2020 national survey on job satisfaction in the network public health institutes of Serbia of the Ministry of Health of Serbia and the Institute of Public Health of Serbia “Dr Milan Jovanović Batut” (IPHS). The survey was conducted during a working day in December 2020 among employees who were present at work (i.e., not absent due to fieldwork, training, on-call duty, being temporarily transferred to another job or position, on paid leave, or for any other reason), and voluntarily agreed to participate in the survey. In total, 1663 questionnaires were distributed to 25 IPHs at the national, district, and city levels [48], and all were returned upon completion. The IPHS stored the collected responses in a central electronic database. This number of completed questionnaires corresponds to 55.6% of the total staff of IPHs [49]. The study population (i.e., PHWs) consists of medical doctors (e.g., specialists or with an M.Sc. or a Ph.D. degree in social medicine, epidemiology, hygiene, human ecology, and microbiology), nurses/health technicians, health associates, and administrative and technical workers.

### 2.2. Ethical Consideration

The study complies with the protocol, the instruments, and the methodological guidance of the IPHS [50]. The Ethics Committee of the IPHS approved using the data from the 2020 national survey on job satisfaction in the network public health institutes (Approval No. 2892/1 from 14 May 2021).

### 2.3. Data Source and Variables

The source of the respondent’s data is the electronic database of IPHS. A total of 21 variables includes individual characteristics (six variables), job-related variables (fourteen variables), and the outcome variable of interest (the intention to leave a current job within the next five years).

Six individual variables in the study were the following: age of the respondents (i.e., less than 35 years, from 35 to 54 years, and 55 years and more); gender (male or female); occupation (i.e., physician, dentist, pharmacist, nurse/health technician, health associate, administrative and technical worker); managerial position (yes vs. no); having an additional practice (yes vs. no, where “yes” encompasses “yes, in education”, “yes, in the private health sector” and “yes, in a sector other than healthcare”); and working in the COVID-19 zone (yes vs. no). An additional variable relates to the level of the IPHs (national, district, and city level). The variable “occupation” was re-coded into physician, nurse/health technician, health associate, and administrative and technical worker due to the small number of dentists and pharmacists added to physicians.

The first set of job-related variables report on the “feeling of tension, stress, or pressure while performing the job” in non-pandemic conditions and during the COVID-19 pandemic on a five-point Likert scale (from 1—not at all to 5—very much). The second set describes the job-related challenges during the COVID-19 pandemic, such as work in entirely new circumstances, exhaustion due to the volume of work, exhaustion due to work under personal protective equipment, availability of personal protective equipment, availability of information, uncertainty and fear of infection, and coping with patient experiences. The next set describes the level of overall job satisfaction and satisfaction with the adequacy of work equipment, adequacy of workspace, availability of working time, job autonomy, respect and valuation of the work, cooperation with colleagues, opportunities for professional development and continuing education, financial compensation for work, institutional management and organization, and implementation of adequate measures to prevent and control the spread of COVID-19 infection. Responses on a five-point Likert scale from 1—very dissatisfied to 5—very satisfied were re-coded into 1—dissatisfied (1—very dissatisfied and 2—dissatisfied), 2—neither satisfied nor dissatisfied, and 3—satisfied (4—satisfied, 5—very satisfied) to simplify the interpretation.

Intention to leave a job was the outcome variable of interest in the study. Respondents reported their plans and reasons to leave their current job within the next five years. The response options were: 1—yes, to work in the private health sector; 2—yes, to work in the sector other than health care; 3—yes, to work abroad, or 4—no. The outcome variable has the following answers, “yes” (1—yes, to work in the private health sector; 2—yes, to work in the sector other than health care; 3—yes, to work abroad), and “no” (4—no). The outcome variable was recoded into a dichotomous variable with two potential answers, “yes” which was coded as 1 and encompassed the all the “yes” answers (“yes, to work in the private health sector”; “yes, to work in the sector other than health care”; “yes, to work abroad”), and “no” which was coded as 0 (encompassed the item “no”). “No” was observed as a reference category within the logistic regression models to explore the association of individual and job-related variables with the outcome variable, i.e., intention to leave a job.

### 2.4. Dealing with Missing Data and the Extent of Missingness

We adopted the approach that the best possible method of handling the missing data was to prevent the problem by planning the study well and collecting the data carefully [51]. Therefore, we used data from the routine national study, more precisely, the national survey on job satisfaction in the network public health institutions of Serbia, which is conducted every year by the IPHS and supported by the Ministry of Health of Serbia. According to their protocol and the methodological guidance [50], the survey in 2020 was conducted during one working day among employees who were currently present at work and who wanted to participate in the survey. Although all distributed questionnaires were returned, not all respondents fully completed the questionnaire, which caused missing data, ranging from 1.1% (for the questions “feeling of tension, stress or pressure while performing the job in non-pandemic conditions” and “satisfaction with adequacy of workspace”) to 11.4% (for the question “working in the COVID zone”). The range of the frequencies of missing values is listed below Tables 1 and 2. Many techniques are suggested to minimize the amount of missing data in the clinical research [52]. We assumed that the best approach is to present the missing data in our study, and to perform the logistic regression model using only the data of fully completed questionnaires (models refer to 1151 of 1663, i.e., 69% of the study population).

### 2.5. Statistical Analysis

The study variables were presented as absolute and relative numbers with a 95% confidence interval (95%CI). Both Pearson’s chi-squared test and Mann–Whitney test were used to test for the association between individual and job-related variables regarding the intention to leave a current job. Univariate regression analysis tested the significance of the associations between potential explanatory variables and intention to leave a current job as the study outcome variable. All variables found to be significantly associated with intention to leave a job in the univariate analysis have been included in the multivariate logistic regression model (Table S1).

The multivariate logistic regression model identified factors that explain respondents’ intention to leave a current job, including their Odds Ratio (OR) with a corresponding 95% confidence interval (95%CI). For categorical variables, the OR is presented concerning a reference category, while for continuous variables, the OR represents the increase in odds of the intention to leave a current job, as an outcome of interest, with every one-unit increase in the input variable. The Hosmer and Lemeshow goodness of fit test was performed to determine how well the model fits the data. Multicollinearity was checked with Variance Inflation Factors (VIF). In addition to VIF, we have performed a Spearman correlation coefficient for job-related variables. Although there were statistically significant positive correlations (as expected), all correlation coefficients had a value less than 0.7 (Table S2). Based on that, we concluded that although there was a correlation between variables, it was within the allowed values for VIF (Table S3). All analyses were conducted using Statistical Package for Social Sciences (SPSS) software (SPSS 23.0 for Windows, SPSS Inc., IBM, Armonk, NY, USA). A *p*-value of less than 0.05 was considered significant. The 95% CI of the outcome of interest according to Clopper-Pearson was obtained by conducting a Binomial test [53,54] in SPSS.

## 3. Results

### 3.1. Individual and Job-Related Characteristics of Public Health Workers in Serbia

In 2020, the majority of the total 1663 respondents were females (73.1%), 35–54 years old (62.8%), nurses/health technicians (36.4%), and with a job at a city-level public health institute (67.8%) (Table 1). Approximately one-fifth (19.4%) of participants were managers, and 10.7% had an additional practice. More than a third (36.4%) of respondents worked in the COVID-19 zone. Almost 40% of respondents, more than in normal conditions, a lot and very much felt tension, stress, or pressure while performing their job during the COVID-19 pandemic. The most frequently reported work-related challenges during the COVID-19 pandemic were completely new circumstances (35.5%), uncertainty and fear of infection (33.3%), and exhaustion due to the volume of work (26.7%). Every ninth respondent (11.1%) was dissatisfied with the job in general (Table 2).

### 3.2. Prevalence of Intention to Leave a Job among Public Health Workers in Serbia

Overall, 20.3% of the respondents were considering leaving their current job within the next five years; 9.8% to work abroad, 8.2% to work in a sector other than healthcare, and 2.3% to work in the private health sector (Table S4).

The respondents who reported intention to leave their current job and those with no such intention significantly differ by gender, age, additional practice, and the majority of job-related characteristics. Among those who significantly more often than their counterparts reported the intention to leave their current job, there were more men (*p* < 0.001), people younger than 55 years (*p* < 0.001), and people who had an additional practice (*p* < 0.001), (Table 1). Intention to leave their current job was higher for respondents who a lot and very much felt tension, stress or pressure while performing the job, both in non-pandemic conditions and during the COVID-19 pandemic, than respondents who only a little or moderately had these feelings, or who did not have such feelings at all (Table 2). Those who considered that exhaustion due to the volume of work, exhaustion due to work under personal protective equipment, and availability of information posed challenges of work during the COVID-19 pandemic significantly more often reported intention to leave their current job, as those who did not feel uncertainty and fear of infection. More than a quarter of respondents (27%) who reported having intention to leave their current job were dissatisfied with their job in general compared to 5.9% of those who did not have such intention (*p* < 0.001). Additionally, the intention to leave their current job was influenced by satisfaction with all examined aspects of work (Table 2).

### 3.3. Potential Predictors of Intention to Leave a Job among Public Health Workers in Serbia

Multivariate analysis revealed several independent predictors of intention to leave their current job among both individual and job-related variables (Table 3).

Males and respondents with an additional practice were by 1.79 and 2.50, respectively, more likely than their counterparts to report an intention to leave a current job. Similarly, respondents younger than 35 years and those aged 35–54 years had 2.97 and 3.28 greater odds, respectively, than respondents older than 55 years to report an intention to leave their current job.

Every unit of increase in the feeling of tension, stress, or pressure while performing the job during the COVID-19 pandemic resulted in increasing the odds of reporting intention to leave a current job by 30%. Unavailability of information and uncertainty and fear of infection were challenges for work during the COVID-19 pandemic, which by 1.93 times (more likely) and by 45% (less likely), respectively, predicted intention to leave a current job. Respondents generally dissatisfied with their job and those who were neither satisfied nor dissatisfied had 3.27 and 2.39 times, respectively, greater odds to have an intention to leave their current job than those who were satisfied. Additionally, those dissatisfied with respect and valuation of their work and those who were neither satisfied nor dissatisfied with financial compensation for work had 3.23 and 1.69 times, respectively, greater odds of reporting such intention than those who were satisfied (Table 3, Figure S1).

## 4. Discussion

According to our knowledge, this was the first study that investigated Serbian PHWs’ intention to leave their current job during the pandemic. Slightly over a fifth of PHWs in Serbia reported intention to leave their current job within the next five years. Both individual and job-related characteristics of respondents were revealed as predictors of intention to leave a current job. Males and persons younger than 55 years and those who had an additional practice were more likely to intend to leave their current job within the next five years than their counterparts. Feelings of job-related tension, stress, or pressure, as well as availability of information during the COVID-19 pandemic also predicted public health workers’ intentions to leave a current job. Conversely, perception of uncertainty and fear of infection as a work challenge during the COVID-19 pandemic predicted lower such intention. Respondents dissatisfied with their job in general and with respect and valuation of their work had greater odds to report the intention to leave their current job, as were those who were neither satisfied nor dissatisfied with financial compensation for work.

In comparison with developed economies where high intention to leave and retire is foreseen by 2023 [42,44], in Serbia, a smaller percentage of PHWs intend to leave their job by 2025, even less than all health workers [55].

Our study has demonstrated that males are more likely to report an intention to leave than females, which is in concordance with previous studies in public health [41] and in other fields [28,29,56,57]. There is empirical evidence that higher job satisfaction and lower job expectations are relevant explanations of gender differences in the intention to leave, making females less willing to leave their current job [56,57,58]. Female PHWs, especially those with children, are probably aware of the benefits offered by working in the field of public health, i.e., having a stable job with clearly defined working hours, not burdened with an emotional toll [59].

As in our study, the literature shows that younger health professionals are more mobile and willing to change jobs while looking for opportunities to improve their education and career [59]. In Serbia, an additional problem is a declining trend in the share of public health professionals under 35, which means that they are not sufficient in numbers to replace older ones who plan to retire or intend to leave a job [31]. Additionally, an important issue is how attractive a job in public health may be for young professionals [59]. The management interventions to recruit and retain the next generation of PHWs should orient towards supportive strategies of innovation and creativity [43,45], including offering additional professional opportunities.

Our study showed that PHWs with an additional practice were 2.5 times more likely to report an intention to leave a current job than their counterparts. It may be that having an additional practice in Serbia represents a ‘stepping stone’ for public health workforce turnover. The study findings are in line with Mobley’s theory that the existence of good alternatives facilitates actually leaving the current job [20]. While some want an additional practice as an income-generating source, a double workload is a push factor to leave for others [57,60]. This finding is essential to be further studied because almost every fifth potential leaver among Serbian PHWs has an additional practice. Study evidence contributes to creating retention strategies to secure the availability of a sufficient public health workforce for the transfer of knowledge and skills from more experienced workers to others and maintain the standard of quality of services.

When health workers experience more dynamic workloads and work-related stress, high intention to leave their current job is related [28,44,61]. Our study confirmed these findings, as increasing the feeling of job-related tension, stress, or pressure during the COVID-19 pandemic results in a significant increase in the likelihood of intention to leave a job among Serbian PHWs. This is an important finding, indicating a need for mental health and psychological support for PHWs during pandemics [61].

Even though COVID-19 is a novel infectious disease, PHWs have been expected to be knowledge brokers about the origin of the virus, models of its transmission, and prevention and control measures (including vaccines) [62]. Our study pointed out that insufficient information about COVID-19 was a challenge for Serbian PHWs and an even greater predictor for leaving their job. This finding calls for better management of risk and crisis communication and thus helps prevent PHWs’ turnover.

PHWs’ training includes preparedness for dealing with outbreaks of infectious disease. Therefore, uncertainty and fear of infection during the COVID-19 pandemic negatively predicted PHWs’ intention to leave their job.

As in previous studies, in our study, job dissatisfaction, in general, was associated with higher intention to leave one’s job [28,29,30,40,43,57], as was dissatisfaction with respect and valuation of one’s work [43,56]. Pay satisfaction is among the most critical aspects of health workers’ retention [39,40,44]. However, as for US government PHWs [63], for Serbian PHWs, the pay was not the most influential factor for leaving the job. People probably decide to leave regardless of the so-called “COVID supplement”, such as salary increases or bonuses for a certain period [64]. Therefore, further research is needed to indicate a payment approach that prevents the intention to leave the current job in public health institutes.

It is important to take the broader context into consideration while discussing issues of recruitment and retention within the public health field. Direct comparisons of our results to other studies were difficult due to methodological differences (e.g., the use of different instruments, various definition of the public health workers, skill-mix and competencies) [40,65] or the characteristics of the labor market and organizational culture [63]. With regard to the latter, it was found that shorter employment in their current position is associated with a higher chance of leaving an organization [40], being dissatisfied with the job, perceiving a lack of organizational support, and burnout were associated with higher odds of intent to leave [45], while gaining at least one promotion is associated with not thinking about leaving [65]. Using different study instruments, other authors found that significant predictors of lower intent to leave the job are greater employee engagement, organizational support, job satisfaction [40], organization satisfaction, pay satisfaction [40,65], job security, and competitive benefits [63].

The current study should be interpreted with several limitations in mind. First, due to the cross-sectional design, we could not determine the causal relationship between the variables. Instead, we identified potential predictors of PHWs’ intention to leave their current job. Second, it is possible that survey data was burdened with nonresponse bias since the study captured respondents who were at work at the time of the survey and those who were willing to participate. During the COVID-19 pandemic, PHWs who were redeployed or worked remotely were more likely to be absent from work. The potential significant difference between respondents and nonrespondents can lead to an over or underestimation of the study findings. Since they work in small teams, perhaps PHWs had concerns about the privacy and confidentiality of the data. That is probably the reason why some variables have missing data (ranging from 1.1 to 11.4%). We did not examine the actual leaving of a job among PHWs. Instead, we explored its strong predictor of leaving a current job [19,20]. In line with the IPHS’s protocol of the 2020 national survey on job satisfaction [50], the single questionnaire was distributed to all workers in the Serbian public healthcare sector. Therefore, some questions were not fully tailored to or related to PHWs. In the future, the creation of questionnaires that are specific to PHWs should be considered.

The COVID-19 pandemic has impacted health workers across the globe, changing the way they work and live, causing uncertainty regarding workplace safety, fear for personal/family health, sense of unpreparedness for additional job responsibilities, lack of understanding of the importance of one’s role during a pandemic, and making issues regarding commitment to the job even more complicated [66,67,68]. Strengthening the PHWs’ preparedness and capacity in the long run after the pandemic requires implementation of various retention strategies. This study highlights all aspects that can influence PHWs’ intention to leave a job. In addition to providing training to help them do their job with more confidence, public health managers could implement measures such as: ensuring availability of psychological counseling, helplines, and social media platforms, introducing flexibility in work (or remote work) for particular profiles of PHWs, as well as leisure activities and motivational workshops and sessions [69]. Concrete retention strategies include fostering a supportive work environment, explicit sharing of responsibility, improving reward systems, ensuring recognition and appreciation of one’s work to effectively increase job satisfaction. Value-based visionary and organizational leadership in public health careers and regulatory interventions, teamwork, symmetry and transparency in everyday communication practices cultivate employee trust, satisfaction, and a sense of empowerment and could contribute employee retention [70,71].

## 5. Conclusions

This study revealed eight predictors of Serbian PHWs’ intention to leave the job, including the following: male gender, younger age, having an additional practice, feelings of job-related tension, stress, or pressure, as well as availability of information during the COVID-19 pandemic, dissatisfaction with a job in general, particularly with respect and valuation of one’s work. In contrast, uncertainty, and fear of infection during the COVID-19 pandemic are inversely associated with such intention. Effective strategies to promote retention, such as health workforce development planning, career and regulatory interventions, and occupational safety and health programs in public health organizations should include measures to increase job satisfaction, transparent and timely sharing of information, reductions in job-related stress, and recognition of PHWs’ value and achievements.

## Figures and Tables

**Table 1:** Individual characteristics of public health workers from 25 public health institutes (*n* = 1663), according to their intention to leave a current job within the next five years, Serbia, 2020.

	Total Public Health Workers		Have Intention to Leave a Current Job		Does Not Have Intention to Leave a Current Job		
Individual Characteristics	*n*	Prevalence (95%CI)	*n*	Prevalence (95%CI)	*n*	Prevalence (95%CI)	*p* Value
Gender, total (*n*)	1508		281		1186		
Male	405	26.9 (24.6–29.1)	109	38.8 (33.1–44.5)	284	23.9 (21.5–26.4)	**<0.001**
Female	1103	73.1 (70.9–75.4)	172	61.2 (55.5–66.9)	902	76.1 (73.6–78.5)	
Age, total (*n*)	1554		295		1219		
<35 years	224	14.4 (12.7–16.2)	43	14.6 (10.5–18.6)	174	14.3 (12.3–16.2)	**<0.001** *
35–54 years	975	62.8 (60.3–65.5)	218	73.9 (68.9–78.9)	730	59.9 (57.1–62.6)	
>55 years	355	22.8 (20.7–24.9)	34	11.5 (7.9–15.2)	315	25.8 (23.4–28.3)	
Occupation, total (*n*)	1500		269		1192		
Physician	323	21.5 (19.4–23.6)	54	20.1 (15.3–24.9)	261	21.9 (19.5–24.2)	0.841
Nurse/health technician	545	36.4 (33.9–38.8)	100	37.2 (31.4–43.0)	432	36.2 (33.5–39.0)	
Health associate	374	24.9 (22.7–27.1)	71	26.4 (21.1–31.7)	293	24.6 (22.1–27.0)	
Administrative worker	168	11.2 (9.6–12.8)	31	11.5 (7.7–15.4)	132	11.1 (9.3–12.9)	
Technical worker	90	6.0 (4.8–7.2)	13	4.8 (2.2–7.4)	74	6.2 (4.8–7.6)	
Managerial position, total (*n*)	1516		286		1191		
Yes	294	19.4 (17.4–21.4)	55	19.2 (14.6–23.8)	233	19.6 (17.3–21.8)	0.899
No	1222	80.6 (78.6–82.6)	231	80.8 (76.2–85.4)	958	80.4 (78.2–82.7)	
Level of the public health institution, total (*n*)	1663		320		1258		
National	89	5.4 (4.3–6.4)	19	5.9 (3.3–8.5)	62	4.9 (3.7–6.1)	0.065
District	446	26.8 (24.7–28.9)	71	22.2 (17.6–26.8)	360	28.6 (26.1–31.1)	
City	1128	67.8 (65.6–70.1)	230	71.9 (66.9–76.8)	836	66.5 (63.8–69.1)	
Having an additional practice, total (*n*)	1663		320		1258		
Yes	178	10.7 (9.2–12.2)	66	20.6 (16.2–25.1)	109	8.7 (7.1–10.2)	**<0.001**
No	1485	89.3 (87.8–90.8)	254	79.4 (74.9–83.8)	1149	91.3 (89.8–92.9)	
Working in the COVID zone, total (*n*)	1474		281		1151		
Yes	536	36.4 (33.9–38.8)	110	39.1 (33.4–44.9)	408	35.4 (32.7–38.2)	0.247
No	938	63.6 (61.2–66.1)	171	60.9 (55.1–66.6)	743	64.6 (61.8–67.3)	

Missing data per variable (range, 6.6–11.4%); *n*—number of respondents; CI—confidence interval; Significant findings according to Pearson chi-square test and Mann–Whitney test * (*p* < 0.05) are marked in bold.

**Table 2:** Job-related characteristics of public health workers from 25 public health institutes (*n* = 1663), according to their intention to leave a current job within the next five years, Serbia, 2020.

	Total Public Health Workers		Have Intention to Leave a Current Job		Does Not Have Intention to Leave a Current Job		
Job-Related Characteristics	*n*	Prevalence (95%CI)	*n*	Prevalence (95%CI)	*n*	Prevalence (95%CI)	*p* Value
Feeling of tension, stress, or pressure while performing the job							
(a) in non-pandemic conditions, total (*n*)	1645		317		1249		
Not at all	185	11.3 (9.7–12.8)	14	4.4 (2.1–6.7)	167	13.4 (11.5–15.3)	**<0.001** *
A little	282	17.1 (15.3–19.0)	37	11.7 (8.1–15.2)	236	18.9 (16.7–21.1)	
Moderately	673	40.9 (38.5–43.3)	111	35.0 (29.7–40.3)	534	42.7 (40.0–45.5)	
A lot	313	19.0 (17.1–20.9)	89	28.1 (23.1–33.0)	207	16.6 (14.5–18.6)	
Very much	192	11.7 (10.1–13.2)	66	20.8 (16.3–25.3)	105	8.4 (6.9–9.9)	
(b) during the COVID-19 pandemic, total (*n*)	1630		313		1240		
Not at all	127	7.8 (6.5–9.1)	10	3.2 (1.2–5.1)	115	9.3 (7.7–10.9)	**<0.001** *
A little	221	13.5 (11.9–15.2)	26	8.3 (5.2–11.4)	186	15.0 (13.0–17.0)	
Moderately	585	35.9 (33.6–38.2)	94	30.0 (24.9–35.1)	471	38.0 (35.3–40.7)	
A lot	394	24.2 (22.1–26.2)	83	26.6 (21.6–31.4)	289	23.3 (20.9–25.7)	
Very much	303	18.6 (16.7–20.5)	100	31.9 (26.7–37.1)	179	14.4 (12.5–16.4)	
The job-related challenges during the COVID-19 pandemic							
(a) work in entirely new circumstances, total (*n*)	1663		320		1258		
Yes	590	35.5 (33.2–37.8)	101	31.6 (26.4–36.7)	473	37.6 (34.9–40.3)	**0.045**
No	1073	64.5 (62.2–66.8)	219	68.4 (63.3–73.6)	785	62.4 (59.7–65.1)	
(b) exhaustion due to the volume of work, total (*n*)	1663		320		1258		
Yes	444	26.7 (24.6–28.8)	113	35.3 (30.0–40.6)	312	24.8 (22.4–27.2)	**<0.001**
No	1219	73.3 (71.2–75.4)	207	64.7 (59.4–69.9)	946	75.2 (72.8–77.6)	
(c) exhaustion due to work under PPE, total (*n*)	1663		320		1258		
Yes	351	21.1 (19.1–23.1)	83	25.9 (21.1–30.8)	262	20.8 (18.6–23.1)	**0.048**
No	1312	78.9 (76.9–80.9)	237	74.1 (69.2–78.9)	996	79.2 (76.9–81.4)	
(d) availability of PPE, total (*n*)	1663		320		1258		
Yes	143	8.6 (7.2–9.9)	36	11.3 (7.8–14.7)	105	8.3 (6.8–9.9)	0.104
No	1520	91.4 (90.0–92.7)	284	88.7 (85.3–92.2)	1153	91.7 (90.1–93.2)	
(e) availability of information, total (*n*)	1663		320		1258		
Yes	266	16.0 (14.2–17.8)	85	26.6 (21.7–31.4)	172	13.7 (11.8–15.6)	**<0.001**
No	1397	84.0 (82.2–85.8)	235	73.4 (68.6–78.3)	1086	86.3 (84.4–88.2)	
(f) uncertainty, and fear of infection, total (*n*)	1663		320		1258		
Yes	554	33.3 (31.0–35.6)	88	27.5 (22.6–32.4)	452	35.9 (33.3–38.6)	**0.005**
No	1109	66.7 (64.4–68.9)	232	72.5 (67.6–77.4)	806	64.1 (61.4–66.7)	
(g) coping with patient experiences, total (*n*)	1663		320		1258		
Yes	187	11.2 (9.7–12.8)	34	10.6 (7.2–14.0)	149	11.8 (10.1–13.6)	0.543
No	1476	88.8 (87.2–90.3)	286	89.4 (86.0–92.8)	1109	88.2 (86.4–89.9)	
Satisfaction with							
(a) job in general, total (*n*)	1568		304		1206		
Dissatisfied	173	11.1 (9.5–12.6)	82	27.0 (22.0–32.0)	71	5.9 (4.6–7.2)	**<0.001** *
Neither satisfied nor dissatisfied	372	23.7 (21.6–25.8)	125	41.1 (35.6–46.7)	229	19.0 (16.8–21.2)	
Satisfied	1023	65.2 (62.9–67.6)	97	31.9 (26.6–37.2)	906	75.1 (72.7–77.6)	
(b) adequacy of work equipment, total (*n*)	1637		317		1242		
Dissatisfied	133	8.1 (6.8–9.4)	50	15.8 (11.7–19.8)	68	5.5 (4.2–6.7)	**<0.001** *
Neither satisfied nor dissatisfied	306	18.7 (16.8–20.6)	84	26.5 (21.6–31.4)	201	16.2 (14.1–18.2)	
Satisfied	1198	73.2 (71.0–75.3)	183	57.7 (52.3–63.2)	973	78.3 (76.0–80.6)	
(c) adequacy of workspace, total (*n*)	1645		315		1249		
Dissatisfied	250	15.2 (13.5–16.9)	80	25.4 (20.6–30.2)	150	12.0 (10.2–13.8)	**<0.001** *
Neither satisfied nor dissatisfied	310	18.8 (16.9–20.7)	79	25.1 (20.3–29.9)	212	17.0 (14.9–19.1)	
Satisfied	1085	66.0 (63.7–68.2)	156	49.5 (44.0–55.1)	887	71.0 (68.5–73.5)	
(d) availability of working time, total (*n*)	1640		314		1246		
Dissatisfied	105	6.4 (5.2–7.6)	43	13.7 (9.9–17.5)	43	3.4 (2.4–4.5)	**<0.001** *
Neither satisfied nor dissatisfied	223	13.6 (11.9–15.3)	65	20.7 (16.2–25.2)	139	11.2 (9.4–12.9)	
Satisfied	1312	80.0 (78.1–81.9)	206	65.6 (60.3–70.9)	1064	85.4 (83.4–87.4)	
(e) job autonomy, total (*n*)	1608		310		1222		
Dissatisfied	246	15.3 (13.5–17.1)	86	27.7 (22.7–32.7)	132	10.8 (9.1–12.5)	**<0.001** *
Neither satisfied nor dissatisfied	322	20.0 (18.1–22.0)	89	28.7 (23.6–33.8)	215	17.6 (15.5–19.7)	
Satisfied	1040	64.7 (62.3–67.0)	135	43.6 (38.0–49.1)	875	71.6 (69.1–74.1)	
(f) respect and valuation of the work, total (*n*)	1633		316		1238		
Dissatisfied	304	18.6 (16.7–20.5)	127	40.2 (34.7–45.6)	142	11.5 (9.7–13.2)	**<0.001** *
Neither satisfied nor dissatisfied	283	17.3 (15.5–19.2)	77	24.4 (19.6–29.1)	194	15.7 (13.6–17.7)	
Satisfied	1046	64.1 (61.7–66.4)	112	35.4 (30.1–40.7)	902	72.8 (70.4–75.3)	
(g) cooperation with colleagues, total (*n*)	1632		310		1243		
Dissatisfied	116	7.1 (5.9–8.4)	45	14.5 (10.6–18.5)	58	4.7 (3.5–5.8)	**<0.001** *
Neither satisfied nor dissatisfied	267	16.4 (14.6–18.2)	83	26.8 (21.8–31.7)	165	13.3 (11.4–15.2)	
Satisfied	1249	76.5 (74.5–78.6)	182	58.7 (53.2–64.2)	1020	82.0 (79.9–84.2)	
(h) opportunities for professional developments and CE, total (*n*)	1522		303		1147		
Dissatisfied	288	18.9 (16.9–20.9)	106	35.0 (29.6–40.4)	150	13.1 (11.1–15.0)	**<0.001** *
Neither satisfied nor dissatisfied	315	20.7 (18.7–22.7)	84	27.7 (22.6–32.8)	214	18.7 (16.4–20.9)	
Satisfied	919	60.4 (57.9–62.8)	113	37.3 (31.8–42.8)	783	68.2 (65.6–71.0)	
(i) financial compensation for work, total (*n*)	1625		312		1233		
Dissatisfied	263	16.2 (14.4–18.0)	95	30.5 (25.3–35.6)	138	11.2 (9.4–12.9)	**<0.001** *
Neither satisfied nor dissatisfied	309	19.0 (17.1–20.9)	89	28.5 (23.5–33.6)	200	16.2 (14.2–18.3)	
Satisfied	1053	64.8 (62.5–67.1)	128	41.0 (35.5–46.5)	895	72.6 (70.1–75.1)	
(j) institutional management and organization, total (*n*)	1569		299		1193		
Dissatisfied	301	19.2 (17.2–21.1)	123	41.1 (35.5–46.7)	147	12.3 (10.4–14.2)	**<0.001** *
Neither satisfied nor dissatisfied	307	19.6 (17.6–21.5)	78	26.1 (21.1–31.1)	210	17.6 (15.4–19.8)	
Satisfied	961	61.2 (58.8–63.7)	98	32.8 (27.4–38.1)	836	70.1 (67.5–72.7)	
(k) preventing and controlling the spread of COVID-19 infection, total (*n*)	1621		314		1232		
Dissatisfied	192	11.8 (10.3–13.4)	70	22.3 (17.7–26.9)	103	8.4 (6.8–9.9)	**<0.001** *
Neither satisfied nor dissatisfied	297	18.3 (16.4–20.2)	83	26.4 (21.5–31.3)	197	16.0 (13.9–18.0)	
Satisfied	1132	69.9 (67.6–72.1)	161	51.3 (45.7–56.8)	932	75.6 (73.2–78.0)	

Missing data per variable (range, 1.1–8.5%); *n*—number of respondents; CI—confidence interval; PPE—personal protective equipment; CE—continuing education; Significant findings according to Pearson chi-square test and Mann–Whitney test * (*p* < 0.05) are marked in bold.

**Table 3:** Potential predictors of the intention to leave a current job within the next five years among public health workers from 25 public health institutes, Serbia, 2020.

	Univariate Regression Analysis *n* = 1663		Multivariate Regression Analysis ^a,b,c^ *n* = 1151	
	OR	95% CI	OR	95% CI
**Individual Characteristics**				
Gender				
Male	**2.01**	**1.53–2.65**	**1.79**	**1.25–2.57**
Female	ref		ref	
Age				
<35 years	**2.29**	**1.41–3.72**	**2.97**	**1.50–5.89**
35–54 years	**2.77**	**1.88–4.06**	**3.28**	**1.91–5.65**
>55 years	ref		ref	
Having an additional practice				
Yes	**2.74**	**1.96–3.83**	**2.50**	**1.56–4.00**
No	ref		ref	
**Job-related characteristics**				
Feeling of tension, stress, or pressure while performing the job				
(a) in non-pandemic conditions	**1.67**	**1.49–1.88**	1.01	0.80–1.26
(b) during the COVID-19 pandemic	**1.59**	**1.41–1.79**	**1.30**	**1.05–1.62**
The job-related challenges during the COVID-19 pandemic				
(a) work in entirely new circumstances				
Yes	**0.76**	**0.59–0.99**	0.92	0.63–1.32
No	ref		ref	
(b) exhaustion due to the volume of work				
Yes	**1.65**	**1.27–2.15**	1.44	0.98–2.10
No	ref		ref	
(c) exhaustion due to work under PPE				
Yes	**1.33**	**1.00–1.77 ^d^**	1.03	0.68–1.55
No	ref		ref	
(d) availability of information				
Yes	**2.28**	**1.70–3.07**	**1.93**	**1.27–2.94**
No	ref		ref	
(e) uncertainty, and fear of infection				
Yes	**0.68**	**0.52–0.89**	**0.55**	**0.37–0.81**
No	ref		ref	
Satisfaction with				
(a) job in general				
Dissatisfied	**10.8**	**7.4–15.8**	**3.27**	**1.64–6.52**
Neither satisfied nor dissatisfied	**5.1**	**3.8–6.9**	**2.39**	**1.47–3.89**
Satisfied	ref		ref	
(b) adequacy of work equipment				
Dissatisfied	**3.91**	**2.63–5.82**	0.59	0.28–1.23
Neither satisfied nor dissatisfied	**2.22**	**1.65–3.00**	1.05	0.61–1.79
Satisfied	ref		ref	
(c) adequacy of workspace				
Dissatisfied	**3.03**	**2.20–4.18**	0.79	0.45–1.40
Neither satisfied nor dissatisfied	**2.12**	**1.55–2.89**	1.05	0.63–1.75
Satisfied	ref		ref	
(d) availability of working time				
Dissatisfied	**5.16**	**3.30–8.09**	1.37	0.65–2.88
Neither satisfied nor dissatisfied	**2.41**	**1.74–3.36**	1.09	0.65–1.83
Satisfied	ref		ref	
(e) job autonomy				
Dissatisfied	**4.22**	**3.05–5.85**	0.60	0.31–1.17
Neither satisfied nor dissatisfied	**2.68**	**1.97–3.64**	0.80	0.47–1.33
Satisfied	ref		ref	
(f) respect and valuation of the work				
Dissatisfied	**7.20**	**5.28–9.82**	**3.23**	**1.67–6.26**
Neither satisfied nor dissatisfied	**3.20**	**2.30–4.42**	1.55	0.90–2.66
Satisfied	ref		ref	
(g) cooperation with colleagues				
Dissatisfied	**4.35**	**2.86–6.62**	0.81	0.41–1.59
Neither satisfied nor dissatisfied	**2.82**	**2.07–3.83**	1.18	0.73–1.89
Satisfied	ref		ref	
(h) opportunities for professional developments and CE				
Dissatisfied	**4.90**	**3.56–6.73**	0.97	0.52–1.81
Neither satisfied nor dissatisfied	**2.72**	**1.97–3.75**	0.86	0.51–1.45
Satisfied	ref		ref	
(i) financial compensation for work				
Dissatisfied	**4.81**	**3.49–6.63**	1.38	0.78–2.45
Neither satisfied nor dissatisfied	**3.11**	**2.28–4.25**	**1.69**	**1.05–2.72**
Satisfied	ref		ref	
(j) institutional management and organization				
Dissatisfied	**7.14**	**5.19–9.81**	1.62	0.85–3.09
Neither satisfied nor dissatisfied	**3.17**	**2.27–4.42**	1.59	0.93–2.73
Satisfied	ref		ref	
(k) preventing and controlling the spread of COVID-19 infection				
Dissatisfied	**3.93**	**2.78–5.56**	0.91	0.50–1.66
Neither satisfied nor dissatisfied	**2.45**	**1.80–3.31**	0.95	0.58–1.54
Satisfied	ref		ref	

OR—odds ratios; CI—confidence interval; ref—reference category; PPE—personal protective equipment; CE—continuing education; *n*—number of respondents. Significant findings are marked in bold; all variables significantly associated in univariate analysis were included in multivariate analysis using Entry Method “Enter.” ^a^ Hosmer–Lemeshow test *p* > 0.05 indicate that the model was a good fit. ^b^ The total model was significant (*p* < 0.001) and accounted for 34.0 percent of the variance (Nagelkerke R Square = 0.340). ^c^ The VIF for the independent variables in the model ranged from 1.04 to 2.95, which indicated that the model did not suffer from multicollinearity. ^d^ *p* = 0.048.

## Data Availability

The data presented in this study are available on request from the corresponding author.

## Supplementary Materials

The following are available online at www.mdpi.com/article/10.3390/ijerph182010652/s1, Table S1: Intention to leave the current job within the next five years among public health workers from 25 public health institutes, Serbia, 2020. Univariate regression analysis, Table S2: The Spearman correlation coefficient for job related variables, Table S3: Variance inflation factor (VIF) for a set of multivariate regression variables, Table S4: General characteristics of the study population, i.e., public health workers from 25 public health institutes (*n* = 1663), Serbia, 2020 and Figure S1: Multivariate regression model of the intension to leave the current job within the next five years among public health workers, Serbia, 2020.
