# Supplementary material for: Factors Associated with Intention of Serbian Public Health Workers to Leave the Job: A Cross-Sectional, Population-Based Study
Source: Int J Environ Res Public Health. 2021 Oct 11;18(20):10652. doi: 10.3390/ijerph182010652 (PMC8535250; doi:10.3390/ijerph182010652)
Supplement: Supplementary file 1 [file ijerph-18-10652-s001.zip › ijerph-1335500-supplementary.pdf]

Table S1: Intention to leave the current job within the next five years among public health workers from 25 public health institutes, Serbia, 2020. Univariate regression analysis

	**Univariate regression analysis (n=1,663)**	
**Individual characteristics**	**OR**	**95%CI**
Gender, total (n)		
Male	**2.01**	**1.53-2.65**
Female	ref	
Age, total (n)		
<35 years	**2.29**	**1.41-3.72**
35-54 years	**2.77**	**1.88-4.06**
>55 years	ref	
Occupation, total (n)		
Physician	1.18	0.61-2.75
Nurse/health technician	1.32	0.70-2.47
Health associate	1.38	0.72-2.63
Administrative worker	1.34	0.66-2.71
Technical worker	ref	
Managerial position, total (n)		
Yes	0.98	0.71-1.36
No	ref	
Level of the public health institution, total (n)		
City	0.90	0.53-1.53
District	0.64	0.36-1.14
National	ref	
Having an additional practice, total (n)		
Yes	**2.74**	**1.96-3.83**
No	ref	
Working in the COVID zone, total (n)		
Yes	1.71	0.90-1.53
No	ref	
**Job-related characteristics**	**OR**	**95%CI**
Feeling of tension, stress, or pressure while performing the job		
a) in non-pandemic conditions, total (n)	**1.67**	**1.49-1.88**
b) during the COVID-19 pandemic, total (n)	**1.59**	**1.41-1.79**
The job-related challenges during the COVID-19 pandemic		
a) work in entirely new circumstances, total (n)		
Yes	**0.76**	**0.59-0.99**
No	ref	
b) exhaustion due to the volume of work, total (n)		
Yes	**1.65**	**1.27-2.15**
No	ref	
c) exhaustion due to work under PPE, total (n)		
Yes	**1.33**	**1.00-1.77***
No	ref	
d) availability of PPE, total (n)		
Yes	1.39	0.93-2.08
No	ref	
e) availability of information, total (n)		
Yes	**2.28**	**1.70- 3.07**
No	ref	
f) uncertainty, and fear of infection, total (n)		
Yes	**0.68**	**0.52-0.89**
No	ref	

g) coping with patient experiences, total (n)		
Yes	0.88	0.60-1.31
No	ref	
Satisfaction with		
a) job in general, total (n)		
Dissatisfied	**10.8**	**7.4-15.8**
Neither satisfied nor dissatisfied	**5.1**	**3.8-6.9**
Satisfied	ref	
b) adequacy of work equipment, total (n)		
Dissatisfied	**3.91**	**2.63-5.82**
Neither satisfied nor dissatisfied	**2.22**	**1.65-3.00**
Satisfied	ref	
c) adequacy of workspace, total (n)		
Dissatisfied	**3.03**	**2.20-4.18**
Neither satisfied nor dissatisfied	**2.12**	**1.55-2.89**
Satisfied	ref	
d) availability of working time, total (n)		
Dissatisfied	**5.16**	**3.30-8.09**
Neither satisfied nor dissatisfied	**2.41**	**1.74-3.36**
Satisfied	ref	
e) job autonomy, total (n)		
Dissatisfied	**4.22**	**3.05-5.85**
Neither satisfied nor dissatisfied	**2.68**	**1.97-3.64**
Satisfied	ref	
f) respect and valuation of the work, total (n)		
Dissatisfied	**7.20**	**5.28-9.82**
Neither satisfied nor dissatisfied	**3.20**	**2.30-4.42**
Satisfied	ref	
g) cooperation with colleagues, total (n)		
Dissatisfied	**4.35**	**2.86-6.62**
Neither satisfied nor dissatisfied	**2.82**	**2.07-3.83**
Satisfied	ref	
h) opportunities for professional developments and CE, total (n)		
Dissatisfied	**4.90**	**3.56-6.73**
Neither satisfied nor dissatisfied	**2.72**	**1.97-3.75**
Satisfied	ref	
i) financial compensation for work, total (n)		
Dissatisfied	**4.81**	**3.49-6.63**
Neither satisfied nor dissatisfied	**3.11**	**2.28-4.25**
Satisfied	ref	
j) institutional management and organization, total (n)		
Dissatisfied	**7.14**	**5.19-9.81**
Neither satisfied nor dissatisfied	**3.17**	**2.27-4.42**
Satisfied	ref	
k) preventing and controlling the spread of COVID-19 infection, total (n)		
Dissatisfied	**3.93**	**2.78-5.56**
Neither satisfied nor dissatisfied	**2.45**	**1.80-3.31**
Satisfied	ref	

OR- odds ratios; CI- confidence interval; ref- reference category; PPE- personal protective equipment; CE- continuing education; n- number of respondents.
Significant findings are marked in bold
*p=0.048

Table S2: The Spearman correlation coefficient for job related variables

Correlations

			Feeling of tension, stress, or pressure while performing the job in non-pandemic conditions	Feeling of tension, stress, or pressure while performing the job during the COVID-19 pandemic	Work in entirely new circumstances	Exhaustion due to the volume of work	Exhaustion due to work under PPE	Availability of information	Uncertainty, and fear of infection	Satisfaction with adequacy of work equipment	Satisfaction with adequacy workspace	Satisfaction with availability of working time	Satisfaction with job autonomy	Satisfaction with respect and valuation of the work	Satisfaction with cooperation with colleagues	Satisfaction with professional developments and CE	Satisfaction with financial compensation	Satisfaction with institutional management and organization	Satisfaction with preventing and controlling the spread of COVID-19 infection	Satisfaction with job in general
Spearman's rho	Feeling of tension, stress, or pressure while performing the job in non-pandemic conditions	Correlation Coefficient	1.000	.693**	-.073**	.168**	.049*	.044	.040	-.221**	-.256**	-.324**	-.324**	-.361**	-.251**	-.312**	-.304**	-.313**	-.218**	-.388**
		Sig. (2-tailed)		.000	.003	.000	.047	.074	.101	.000	.000	.000	.000	.000	.000	.000	.000	.000	.000	.000
		N	1645	1628	1645	1645	1645	1645	1645	1622	1631	1625	1594	1617	1618	1508	1609	1557	1611	1554
	Feeling of tension, stress, or pressure while performing the job during the COVID-19 pandemic	Correlation Coefficient	.693**	1.000	-.085**	.219**	.087**	.020	.149**	-.196**	-.227**	-.243**	-.260**	-.280**	-.168**	-.264**	-.252**	-.253**	-.212**	-.309**
		Sig. (2-tailed)	.000		.001	.000	.000	.430	.000	.000	.000	.000	.000	.000	.000	.000	.000	.000	.000	.000
		N	1628	1630	1630	1630	1630	1630	1630	1608	1616	1610	1580	1602	1604	1498	1595	1545	1599	1542
	Work in entirely new circumstances	Correlation Coefficient	-.073**	-.085**	1.000	-.021	-.032	.074**	-.047	.140**	.120**	.064**	.134**	.125**	.111**	.170**	.137**	.144**	.163**	.131**
		Sig. (2-tailed)	.003	.001		.384	.186	.002	.056	.000	.000	.010	.000	.000	.000	.000	.000	.000	.000	.000
		N	1645	1630	1663	1663	1663	1663	1663	1637	1645	1640	1608	1633	1632	1522	1625	1569	1621	1568
	Exhaustion due to the volume of work	Correlation Coefficient	.168**	.219**	-.021	1.000	.171**	.074**	.023	.029	.008	-.101**	.016	-.011	-.016	-.019	-.010	-.029	.030	-.050*
		Sig. (2-tailed)	.000	.000	.384		.000	.002	.342	.235	.730	.000	.534	.653	.509	.469	.685	.258	.225	.046
		N	1645	1630	1663	1663	1663	1663	1663	1637	1645	1640	1608	1633	1632	1522	1625	1569	1621	1568
	Exhaustion due to work under PPE	Correlation Coefficient	.049*	.087**	-.032	.171**	1.000	.084**	.147**	.000	-.021	-.013	.019	-.015	-.039	-.016	.000	-.017	.018	-.025
		Sig. (2-tailed)	.047	.000	.186	.000		.001	.000	.989	.388	.589	.445	.532	.115	.543	.996	.509	.457	.320
		N	1645	1630	1663	1663	1663	1663	1663	1637	1645	1640	1608	1633	1632	1522	1625	1569	1621	1568
	Availability of information	Correlation Coefficient	.044	.020	.074**	.074**	.084**	1.000	.116**	-.042	-.036	-.078**	-.016	-.086**	-.077**	-.086**	-.061*	-.141**	-.067**	-.103**
		Sig. (2-tailed)	.074	.430	.002	.002	.001		.000	.088	.149	.002	.515	.001	.002	.001	.013	.000	.007	.000
		N	1645	1630	1663	1663	1663	1663	1663	1637	1645	1640	1608	1633	1632	1522	1625	1569	1621	1568
	Uncertainty, and fear of infection	Correlation Coefficient	.040	.149**	-.047	.023	.147**	.116**	1.000	.020	.006	-.010	.017	.016	.002	-.047	-.015	-.022	.011	.023
		Sig. (2-tailed)	.101	.000	.056	.342	.000	.000		.418	.821	.693	.502	.523	.924	.066	.552	.383	.644	.359
		N	1645	1630	1663	1663	1663	1663	1663	1637	1645	1640	1608	1633	1632	1522	1625	1569	1621	1568
	Satisfaction with adequacy of work equipment	Correlation Coefficient	-.221**	-.196**	.140**	.029	.000	-.042	.020	1.000	.588**	.429**	.464**	.514**	.372**	.540**	.482**	.507**	.479**	.510**
		Sig. (2-tailed)	.000	.000	.000	.235	.989	.088	.418		.000	.000	.000	.000	.000	.000	.000	.000	.000	.000
		N	1622	1608	1637	1637	1637	1637	1637	1637	1627	1620	1588	1610	1610	1506	1606	1550	1602	1546
	Satisfaction with adequacy workspace	Correlation Coefficient	-.256**	-.227**	.120**	.008	-.021	-.036	.006	.588**	1.000	.435**	.437**	.478**	.337**	.445**	.380**	.454**	.410**	.458**
		Sig. (2-tailed)	.000	.000	.000	.730	.388	.149	.821	.000		.000	.000	.000	.000	.000	.000	.000	.000	.000
		N	1631	1616	1645	1645	1645	1645	1645	1627	1645	1629	1595	1619	1619	1515	1612	1556	1607	1554
	Satisfaction with availability of working time	Correlation Coefficient	-.324**	-.243**	.064**	-.101**	-.013	-.078**	-.010	.429**	.435**	1.000	.485**	.464**	.399**	.416**	.441**	.419**	.334**	.482**
		Sig. (2-tailed)	.000	.000	.010	.000	.589	.002	.693	.000	.000		.000	.000	.000	.000	.000	.000	.000	.000
		N	1625	1610	1640	1640	1640	1640	1640	1620	1629	1640	1592	1618	1616	1509	1611	1554	1603	1550
	Satisfaction with job autonomy	Correlation Coefficient	-.324**	-.260**	.134**	.016	.019	-.016	.017	.464**	.437**	.485**	1.000	.685**	.478**	.569**	.488**	.577**	.433**	.567**
		Sig. (2-tailed)	.000	.000	.000	.534	.445	.515	.502	.000	.000	.000		.000	.000	.000	.000	.000	.000	.000
		N	1594	1580	1608	1608	1608	1608	1608	1588	1595	1592	1608	1587	1586	1497	1579	1530	1580	1518
	Satisfaction with respect and valuation of the work	Correlation Coefficient	-.361**	-.280**	.125**	-.011	-.015	-.086**	.016	.514**	.478**	.464**	.685**	1.000	.557**	.596**	.548**	.671**	.486**	.647**
		Sig. (2-tailed)	.000	.000	.000	.653	.532	.001	.523	.000	.000	.000	.000		.000	.000	.000	.000	.000	.000
		N	1617	1602	1633	1633	1633	1633	1633	1610	1619	1618	1587	1633	1610	1502	1600	1547	1597	1543
	Satisfaction with cooperation with colleagues	Correlation Coefficient	-.251**	-.168**	.111**	-.016	-.039	-.077**	.002	.372**	.337**	.399**	.478**	.557**	1.000	.472**	.403**	.505**	.415**	.498**
		Sig. (2-tailed)	.000	.000	.000	.509	.115	.002	.924	.000	.000	.000	.000	.000		.000	.000	.000	.000	.000
		N	1618	1604	1632	1632	1632	1632	1632	1610	1619	1616	1586	1610	1632	1505	1604	1550	1599	1542
	Satisfaction with professional developments and CE	Correlation Coefficient	-.312**	-.264**	.170**	-.019	-.016	-.086**	-.047	.540**	.445**	.416**	.569**	.596**	.472**	1.000	.622**	.655**	.505**	.585**
		Sig. (2-tailed)	.000	.000	.000	.469	.543	.001	.066	.000	.000	.000	.000	.000	.000		.000	.000	.000	.000
		N	1508	1498	1522	1522	1522	1522	1522	1506	1515	1509	1497	1502	1505	1522	1506	1457	1499	1443
	Satisfaction with financial compensation	Correlation Coefficient	-.304**	-.252**	.137**	-.010	.000	-.061*	-.015	.482**	.380**	.441**	.488**	.548**	.403**	.622**	1.000	.590**	.440**	.583**
		Sig. (2-tailed)	.000	.000	.000	.685	.996	.013	.552	.000	.000	.000	.000	.000	.000	.000		.000	.000	.000
		N	1609	1595	1625	1625	1625	1625	1625	1606	1612	1611	1579	1600	1604	1506	1625	1549	1595	1538
	Satisfaction with institutional management and organization	Correlation Coefficient	-.313**	-.253**	.144**	-.029	-.017	-.141**	-.022	.507**	.454**	.419**	.577**	.671**	.505**	.655**	.590**	1.000	.618**	.645**
		Sig. (2-tailed)	.000	.000	.000	.258	.509	.000	.383	.000	.000	.000	.000	.000	.000	.000	.000		.000	.000
		N	1557	1545	1569	1569	1569	1569	1569	1550	1556	1554	1530	1547	1550	1457	1549	1569	1548	1484
	Satisfaction with preventing and controlling the spread of COVID-19 infection	Correlation Coefficient	-.218**	-.212**	.163**	.030	.018	-.067**	.011	.479**	.410**	.334**	.433**	.486**	.415**	.505**	.440**	.618**	1.000	.514**
		Sig. (2-tailed)	.000	.000	.000	.225	.457	.007	.644	.000	.000	.000	.000	.000	.000	.000	.000	.000		.000
		N	1611	1599	1621	1621	1621	1621	1621	1602	1607	1603	1580	1597	1599	1499	1595	1548	1621	1534
	Satisfaction with job in general	Correlation Coefficient	-.388**	-.309**	.131**	-.050*	-.025	-.103**	.023	.510**	.458**	.482**	.567**	.647**	.498**	.585**	.583**	.645**	.514**	1.000
		Sig. (2-tailed)	.000	.000	.000	.046	.320	.000	.359	.000	.000	.000	.000	.000	.000	.000	.000	.000	.000	
		N	1554	1542	1568	1568	1568	1568	1568	1546	1554	1550	1518	1543	1542	1443	1538	1484	1534	1568

**. Correlation is significant at the 0.01 level (2-tailed).

*. Correlation is significant at the 0.05 level (2-tailed).

PPE- personal protective equipment; CE- continuing education

Table S3: Variance inflation factor (VIF) for a set of multivariate regression variables

Coefficients[a]

Model		Unstandardized Coefficients		Standardized Coefficients	t	Sig.	Collinearity Statistics	
		B	Std. Error	Beta			Tolerance	VIF
1	(Constant)	.712	.098		7.253	.000		
	Feeling of tension, stress, or pressure while performing the job in non-pandemic conditions	-.004	.014	-.012	-.297	.766	.436	2.296
	Feeling of tension, stress, or pressure while performing the job during the COVID-19 pandemic	.034	.013	.099	2.537	.011	.453	2.210
	Work in entirely new circumstances	-.015	.022	-.018	-.677	.498	.924	1.082
	Exhaustion due to the volume of work	.050	.025	.056	2.016	.044	.879	1.137
	Exhaustion due to work under PPE	.003	.026	.003	.125	.901	.940	1.064
	Availability of information	.105	.029	.099	3.664	.000	.934	1.071
	Uncertainty, and fear of infection	-.079	.023	-.093	-3.405	.001	.915	1.092
	Gender	.069	.024	.078	2.910	.004	.959	1.042
	Age	-.053	.018	-.078	-2.929	.003	.965	1.037
	Having an additional practice	.126	.033	.102	3.812	.000	.959	1.042
	Satisfaction with adequacy of work equipment	.032	.025	.048	1.310	.190	.504	1.983
	Satisfaction with adequacy workspace	.010	.019	.019	.553	.580	.595	1.681
	Satisfaction with availability of working time	-.022	.024	-.030	-.908	.364	.638	1.568
	Satisfaction with job autonomy	.038	.022	.067	1.701	.089	.435	2.300
	Satisfaction with respect and valuation of the work	-.098	.024	-.185	-4.118	.000	.339	2.953
	Satisfaction with cooperation with colleagues	.008	.024	.012	.358	.721	.593	1.685
	Satisfaction with professional developments and CE	-.002	.021	-.004	-.102	.919	.414	2.414
	Satisfaction with financial compensation	-.038	.021	-.067	-1.861	.063	.523	1.912
	Satisfaction with institutional management and organization	-.042	.023	-.081	-1.838	.066	.351	2.846
	Satisfaction with preventing and controlling the spread of COVID-19 infection	.017	.021	.029	.819	.413	.550	1.819
	Satisfaction with job in general	-.114	.025	-.187	-4.569	.000	.410	2.441

a. Dependent Variable: intention to leave a job

PPE- personal protective equipment; CE- continuing education

Table S4: General characteristics of the study population i.e. public health workers from 25 public health institutes (n = 1,663), Serbia, 2020.

	Total public health workers	
**Individual characteristics**	**n**	**Prevalence (95%CI)**
Gender, total (n)	1508	
Male	405	26.9 (24.6-29.1)
Female	1103	73.1 (70.9-75.4)
Age, total (n)	1554	
<35 years	224	14.4 (12.7-16.2)
35-54 years	975	62.8 (60.3-65.5)
>55 years	355	22.8 (20.7-24.9)
Occupation, total (n)	1500	
Physician	323	21.5 (19.4-23.6)
Nurse/health technician	545	36.4 (33.9-38.8)
Health associate	374	24.9 (22.7-27.1)
Administrative worker	168	11.2 (9.6-12.8)
Technical worker	90	6.0 (4.8-7.2)
Managerial position, total (n)	1516	
Yes	294	19.4 (17.4-21.4)
No	1222	80.6 (78.6-82.6)
Level of the public health institution, total (n)	1663	
National	89	5.4 (4.3-6.4)
District	446	26.8 (24.7-28.9)
City	1128	67.8 (65.6-70.1)
Having an additional practice, total (n)	1663	
Yes	178	10.7 (9.2-12.2)
No	1485	89.3 (87.8-90.8)
Working in the COVID zone, total (n)	1474	
Yes	536	36.4 (33.9-38.8)
No	938	63.6 (61.2-66.1)
Intentions to leave a current job within next five years, total (n)	1578	
Yes, to work in the private health sector	36	2.3 (1.5-3.0)
Yes, to work in the sector other than healthcare	130	8.2 (6.9-9.6)
Yes, to work abroad	154	9.8 (8.3-11.2)
No	1258	79.7 (77.7-81.7)
**Job-related characteristics**	**n**	**Prevalence (95%CI)**
Feeling of tension, stress, or pressure while performing the job		
a) in non-pandemic conditions, total (n)	1645	
Not at all	185	11.3 (9.7-12.8)
A little	282	17.1 (15.3-19.0)
Moderately	673	40.9 (38.5-43.3)
A lot	313	19.0 (17.1-20.9)
Very much	192	11.7 (10.1-13.2)
b) during the COVID-19 pandemic, total (n)	1630	
Not at all	127	7.8 (6.5-9.1)
A little	221	13.5 (11.9-15.2)
Moderately	585	35.9 (33.6-38.2)
A lot	394	24.2 (22.1-26.2)
Very much	303	18.6 (16.7-20.5)
The job-related challenges during the COVID-19 pandemic		
a) work in entirely new circumstances, total (n)	1663	
Yes	590	35.5 (33.2-37.8)
No	1073	64.5 (66.2-66.8)
b) exhaustion due to the volume of work, total (n)	1663	
Yes	444	26.7 (24.6-28.8)
No	1219	73.3 (71.2-75.4)

c) exhaustion due to work under PPE, total (n)	1663	
Yes	351	21.1 (19.1-23.1)
No	1312	78.9 (76.9-80.9)
d) availability of PPE, total (n)	1663	
Yes	143	8.6 (7.2-9.9)
No	1520	91.4 (90.0-92.7)
e) availability of information, total (n)	1663	
Yes	266	16.0 (14.2-17.8)
No	1397	84.0 (82.2-85.8)
f) uncertainty, and fear of infection, total (n)	1663	
Yes	554	33.3 (31.0-35.6)
No	1109	66.7 (64.4-68.9)
g) coping with patient experiences, total (n)	1663	
Yes	187	11.2 (9.7-12.8)
No	1476	88.8 (87.2-90.3)
Satisfaction with		
a) job in general, total (n)	1568	
Dissatisfied	173	11.1 (9.5-12.6)
Neither satisfied nor dissatisfied	372	23.7 (21.6-25.8)
Satisfied	1023	65.2 (62.9-67.6)
b) adequacy of work equipment, total (n)	1637	
Dissatisfied	133	8.1 (6.8-9.4)
Neither satisfied nor dissatisfied	306	18.7 (16.8-20.6)
Satisfied	1198	73.2 (71.0-75.3)
c) adequacy of workspace, total (n)	1645	
Dissatisfied	250	15.2 (13.5-16.9)
Neither satisfied nor dissatisfied	310	18.8 (16.9-20.7)
Satisfied	1085	66.0 (63.7-68.2)
d) availability of working time, total (n)	1640	
Dissatisfied	105	6.4 (5.2-7.6)
Neither satisfied nor dissatisfied	223	13.6 (11.9-15.3)
Satisfied	1312	80.0 (78.1-81.9)
e) job autonomy, total (n)	1608	
Dissatisfied	246	15.3 (13.5-17.1)
Neither satisfied nor dissatisfied	322	20.0 (18.1-22.0)
Satisfied	1040	64.7 (62.3-67.0)
f) respect and valuation of the work, total (n)	1633	
Dissatisfied	304	18.6 (16.7-20.5)
Neither satisfied nor dissatisfied	283	17.3 (15.5-19.2)
Satisfied	1046	64.1 (61.7-66.4)
g) cooperation with colleagues, total (n)	1632	
Dissatisfied	116	7.1 (5.9-8.4)
Neither satisfied nor dissatisfied	267	16.4 (14.6-18.2)
Satisfied	1249	76.5 (74.5-78.6)
h) opportunities for professional developments and CE, total (n)	1522	
Dissatisfied	288	18.9 (16.9-20.9)
Neither satisfied nor dissatisfied	315	20.7 (18.7-22.7)
Satisfied	919	60.4 (57.9-62.8)
i) financial compensation for work, total (n)	1625	
Dissatisfied	263	16.2 (14.4-18.0)
Neither satisfied nor dissatisfied	309	19.0 (17.1-20.9)
Satisfied	1053	64.8 (62.5-67.1)
j) institutional management and organization, total (n)	1569	
Dissatisfied	301	19.2 (17.2-21.1)
Neither satisfied nor dissatisfied	307	19.6 (17.6-21.5)
Satisfied	961	61.2 (58.8-63.7)
k) preventing and controlling the spread	1621	

of COVID-19 infection, total (n)		
Dissatisfied	192	11.8 (10.3-13.4)
Neither satisfied nor dissatisfied	297	18.3 (16.4-20.2)
Satisfied	1132	69.9 (67.6-72.1)

Missing data per variable (range, 1.1%-11.4%); n- number of respondents; CI- confidence interval; PPE- personal protective equipment; CE- continuing education

Figure S1: Multivariate regression model of the intention to leave the current job within the next five years among public health workers, Serbia, 2020

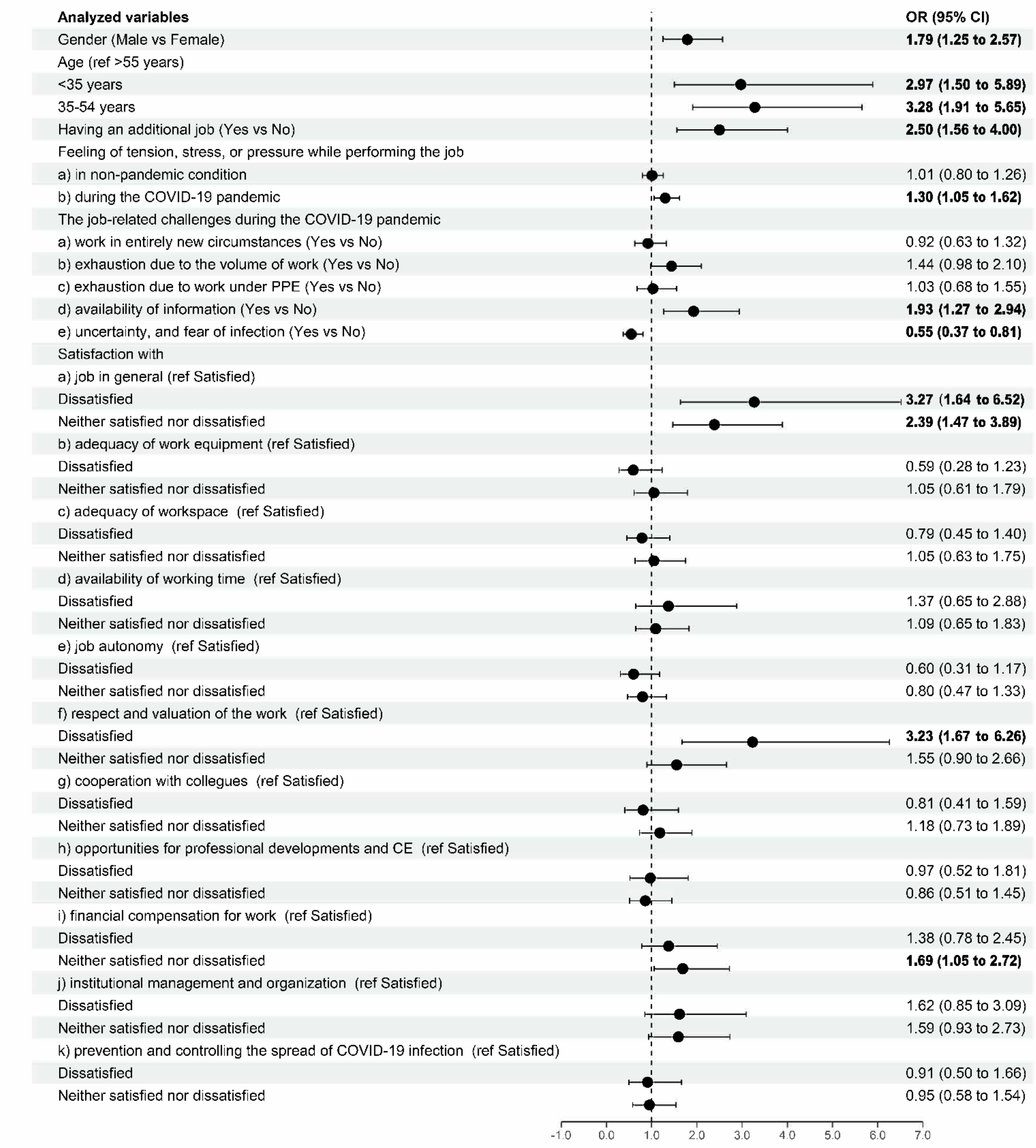

1-reference line representing a likelihood of 100%;
OR>1- likelihood greater than 100%; OR<1- likelihood less than 100%

OR- odds ratios; CI- confidence interval; ref- reference category; PPE- personal protective equipment; CE- continuing education; Significant findings are marked in bold.
